# Hybrid plasmids: genetic diversity and hypervirulent phenotype of *Klebsiella pneumoniae*

**DOI:** 10.3389/fmicb.2026.1768089

**Published:** 2026-04-29

**Authors:** Svetlana Kovalchuk, Anna Arkhipova, Alexandra Melikhova, Anna Akhmetzyanova, Andrey Vvedensky, Tatiana Savinova, Alina Ivkina, Dmitry Konanov, Danil Krivonos, Denis Mokhirev, Yana Benediktova, Lyudmila Fedorova, Elena Ilina

**Affiliations:** Research Institute for Systems Biology and Medicine, Moscow, Russia

**Keywords:** hybrid plasmids, *Klebsiella pneumoniae*, mouselethality assay, multi-drug resistant, virulence

## Abstract

**Background:**

The emergence of hybrid plasmids carrying virulence and multidrug resistance genes in *Klebsiella pneumoniae* can pose a serious public health challenge. However, data on their association with the hypervirulent phenotype of *Klebsiella pneumoniae* are contradictory.

**Methods:**

We performed whole-genome sequencing of 32 *K. pneumoniae* isolates recovered from patients and environmental surfaces in eight Russian hospitals. The virulence of 7 isolates was assessed using a mouse lethality assay. We conducted cluster analysis of the global population of hybrid plasmids from *K. pneumoniae* (*n* = 295) based on their nucleotide sequences.

**Results:**

Whole-genome sequencing showed that the majority of isolates (27/32) carried hybrid plasmids (~272–476 kb), which simultaneously harboring virulence genes (*iucABCD*, *iutA*, and *rmpA2*), and antibiotic resistance genes (ARGs), including *bla_OXA-48_*. The LD_50_ values for the tested isolates were > 10^6^ CFU, a dosage comparable to classical *K. pneumoniae*. Analysis of the global population of hybrid plasmids identified 8 clusters, that showed concordance with replicon type, ARGs, and virulence genes. The majority of the *K. pneumoniae* isolates carrying hybrid plasmids belonged to ST147, ST395, and ST11.

**Conclusion:**

Data regarding the hypervirulence of convergent of *K. pneumoniae* isolates are contradictory. Further studies are needed to understand the genetic basis of the differences in virulence among convergent *K. pneumoniae* isolates, carrying hybrid plasmids.

## 1 Introduction

*Klebsiella pneumoniae*, a clinically significant Gram-negative bacterium, is the third leading cause of mortality from various infectious diseases, including nosocomial pneumonia, urinary tract infections, bacteremia, and liver abscesses (Antimicrobial Resistance Collaborators, 2022). Historically, *K. pneumoniae* has been categorized into two groups: classical (cKp) and hypervirulent *K. pneumoniae* (hvKp) strains. cKp strains are generally of low virulence but are often multidrug-resistant (MDR) (Russo et al., 2024a). In contrast, hvKp strains are typically susceptible to antibiotics but cause severe and invasive community-acquired infections in healthy individuals (Marr and Russo, 2019; Choby et al., 2020; Liu et al., 2020; Kochan et al., 2023; Mendes et al., 2023; Russo et al., 2024a).

For a long time, classical and hypervirulent *K. pneumoniae* (cKp and hvKp) were regarded as distinct clonal lineages. This view changed with the discovery of *K. pneumoniae* isolates exhibiting both MDR and hypervirulence phenotypes, which were designated as convergent isolates. From a genetic point of view, two types of convergent *K. pneumoniae* isolates have been described. The first carries virulence genes (such as *iuc*, *iro*, *rmpA*, and *rmpA2*) and ARGs on separate plasmids, whereas the second contains a hybrid plasmid that harbors both ARGs and the virulence genes (Tang et al., 2020).

Firstly, convergent *K. pneumoniae* isolates were identified in hospitals in China (Yao et al., 2015; Gu et al., 2018). These isolates carried plasmids harboring antibiotic resistance genes (ARGs), including *bla*_KPC-2_, and pLVPK-like 170 kbp plasmids harboring various combinations of virulence genes, such as *iucA*, *rmpA*, *rmpA2*, and *iroN* (Yao et al., 2015; Gu et al., 2018). To date, the distribution of the convergent *K. pneumoniae* isolates has been observed in different geographical regions (Yao et al., 2015; Gu et al., 2018; Lev et al., 2018; Wyres et al., 2020; Volozhantsev et al., 2020; Lazareva et al., 2020; Banerjee et al., 2021; Starkova et al., 2021; Yong et al., 2022; Kochan et al., 2022; Arena et al., 2022; Samoilova et al., 2024; Shapovalova et al., 2024). A recent large-scale analysis showed that the proportion of the convergent *K. pneumoniae* increased from 2% in 2012 to 46% in 2021 (Spadar et al., 2023). The convergent *K. pneumoniae* isolates carrying hybrid plasmids play a critical role in the dissemination of antibiotic resistance genes and virulence factors. However, data regarding the association between the presence of hybrid plasmids and the hypervirulent phenotype in *K. pneumoniae* are still limited and contradictory.

This study aimed to characterize convergent *K. pneumoniae* isolates collected from eight Russian hospitals (2019–2024) and to analyze the diversity of gene content among hybrid plasmids circulating in the global *K. pneumoniae* population.

## 2 Methodology

### 2.1 Bacterial isolates, identification, and growth conditions

*Klebsiella pneumoniae* isolates (*n* = 32) were obtained from patient specimens (bronchial lavage, urine, wounds, blood, and sputum) and environmental surfaces (water taps, washbasins, ward door handles, toilets and flush buttons, windowsills, walls, and furniture in the wards) in six hospitals in Moscow (*n* = 29) and two hospitals in Arkhangelsk (*n* = 3) from 2019 to 2024. *K. pneumoniae* isolates were cultured overnight at 37 °C in nutrient containing 18 g/L pancreatic fish meal hydrolysate and 2 g/L sodium chloride broth (GRM broth), purchased from the Center for Applied Microbiology and Biotechnology (Obolensk, Russia). Bacterial species were identified using MALDI-TOF MS (SMART MS 5020, Zhuhai DL Biotech Co., Ltd., Zhuhai City, China).

### 2.2 Antimicrobial susceptibility testing

The susceptibility of the *K. pneumoniae* isolates to 21 antibiotics—ampicillin (AMP), cefazolin (CFZ), cefuroxime (CXM), aztreonam (AZT), gentamicin (GEN), amikacin (AMK), colistin (COL), trimethoprim/sulfamethoxazole (SXT), ciprofloxacin (CIP), chloramphenicol (CHL), tetracycline (TET), piperacillin (PIP), cefotaxime (CTX), ceftazidime (CAZ), cefoperazone (CFP), cefepime (FEP), meropenem (MEM), ertapenem (ETP), tigecycline (TGC), netilmicin (NET), and tobramycin (TOB) was assessed by broth microdilution using MIC G-I and MIC G-II kits (MIKROLATEST®, Czech Republic). The results were interpreted according to the European Committee on Antimicrobial Susceptibility Testing guidelines (EUCAST breakpoints v 15.0). The PK-PD breakpoint of 1 mg/L was adopted for tigecycline.^1^ For tetracycline and cefoperazone, a breakpoint of ≥16 mg/L was adopted according to CLSI guidelines.^2^ Multidrug resistance (MDR) was defined as acquired non-susceptibility to at least one agent in three or more antimicrobial categories (Magiorakos et al., 2012).

### 2.3 Whole-genome sequencing

Genomic DNA was extracted from bacterial cells using the ExtractDNA Kit (Evrogen JSC, Moscow, Russia) and quantified with a NanoDrop One spectrophotometer (Thermo Fisher Scientific, USA). Whole-genome sequencing was performed on the PromethION platform (Oxford Nanopore Technologies, UK). Libraries were prepared using the Ligation Sequencing gDNA Native Barcoding Kit (SQK-NBD114) and loaded onto a PromethION R10 flow cell according to the manufacturer’s protocol. Reads were assembled *de novo* into contigs using Flye v2.9.5. Completeness of the genomes and contamination level were estimated using CheckM2. Genome assemblies were submitted to NCBI GenBank and annotated using the NCBI Prokaryotic Genome Annotation Pipeline (PGAP v.4.1) (Tatusova et al., 2016). Genomes of *K. pneumoniae* isolates were deposited in GenBank under BioProject ID PRJNA1263478 and PRJNA945360; accession numbers GCA_037477765.1; GCA_037477735.1; GCA_037477855.1; GCA_037477795.1; GCA_037477835.1; GCA_037477745.1; GCA_037477755.1; GCA_037477845.1; GCA_037477825.1; GCA_037477725.1; GCA_037477865.1; GCA_037477875.1, BioProject PRJNA1263478 (GCA_051801345.1, GCA_051800935.1, GCA_051800925.1).

### 2.4 Sequence analysis

Sequence types (STs), antibiotic resistance genes (ARGs), and plasmid replicon types were identified using the MLST (Larsen et al., 2012), ResFinder (Bortolaia et al., 2020), AMRFinderPlus (Feldgarden et al., 2021), and PlasmidFinder (Carattoli et al., 2014) tools. The presence of virulence factors was confirmed using the *K. pneumoniae* virulence database at the Pasteur Institute.^4^ K- and O-antigen loci were determined using Kaptive.^5^ Kleborate v3 software was used to evaluate virulence and resistance scores. Kleborate virulence scores (0–5) classify *K. pneumoniae* by assessing acquired virulence loci (yersiniabactin, colibactin, aerobactin, and salmochelin): 0 -none detected; 1 - yersiniabactin (*ybt*) only; 2 - colibactin (*clb*) ± ybt (no aerobactin); 3 - аerobactin (*iuc*) only; 4 -: аerobactin (*iuc*) + yersiniabactin (*ybt*) (no colibactin); 5 - yersiniabactin (*ybt*) + colibactin (*clb*) + aerobactin (*iuc*). Higher scores (4–5) indicate the presence of aerobactin (associated with hypervirulence/hvKp). Isolates were classified as a hypervirulent Kp (hvKp) if they contained either aerobactin (*iuc*), salmochelin (*iro*) or both gene loci (scores 4–5) (Lam et al., 2022).

### 2.5 String test

The string test was performed to detect hypermucoviscosity as previously described (Hadano, 2013). Briefly, isolates were grown overnight at 37 °C on GRM agar. A colony was lifted with a loop to evaluate the formation of a viscous string. A positive result was defined as a string length ≥ 0.5 cm (Hadano, 2013).

### 2.6 Mouse lethality assay

The virulence of *K. pneumoniae* isolates (*n* = 7) was assessed using a mouse lethality assay (Reed and Muench, 1938). Briefly, isolates were grown on GRM agar overnight at 37 °C. Overnight cultures were resuspended in PBS, and then diluted with PBS to 10^2^, 10^4^, 10^6^, and 10^8^ CFU/mL by measuring OD_600_. Inocula sizes were confirmed by plating bacteria for colony enumeration.

Four- to six-week-old male and female ICR (CD − 1) mice (weighing 18–24 g) were used. Groups of mice (*n* = 4 per dose) were infected intraperitoneally with bacterial suspensions containing 10^2^, 10^4^, 10^6^, or 10^8^ CFU.

All animal procedures are performed according to the guidelines of the European Convention for the Protection of Vertebrate Animals Used for Experimental and other Scientific Purposes (ETS 123), Strasbourg, 1986. All procedures were approved by the Research Institute for Systems Biology and Medicine Bioethics Committee (Protocol no. 3).

### 2.7 Cluster analysis of the hybrid plasmids

Hybrid plasmid sequences were retrieved from the NCBI RefSeq database (as of February 28, 2025) using key words “*Klebsiella pneumoniae*” + “plasmid” + “*iucA/iro*.” Only unfragmented plasmids with length from 200Kb to 1000Kb were considered. For short-read assemblies, only ‘circular’ plasmids were used. For long-read assemblies, both ‘linear’ and ‘circular’ plasmids were used. All plasmids found in RefSeq were screened for the presence of ARGs using ResFinder (Bortolaia et al., 2020). Plasmids were classified as hybrids if they contained aerobactin (*iucA*) or salmochelin (*iro*) genes along with ARGs. Cluster analysis was performed using the Mge-cluster tool (Arredondo-Alonso et al., 2023). Phylogenetic trees were built using Fasttree (Price et al., 2010) based on core genome alignment provided by Roary (Page et al., 2015).

## 3 Results

### 3.1 Antibiotic susceptibility testing

The susceptibility profile of 32 *K. pneumoniae* isolates to 21 antimicrobial agents from 11 drug classes was evaluated (Supplementary Table S1).

One isolate (CIB-6) was resistant to only three antibiotics (AMP, CFZ, and CXM), while the remaining 31 were multidrug-resistant. All MDR isolates were resistant to 11 antibiotics, including cephalosporins (cefuroxime, cefoperazone, cefazolin, cefotaxime), ciprofloxacin, ertapenem, aztreonam, tobramycin, ampicillin, piperacillin, and chloramphenicol. Most isolates (80.6–93.5%) were resistant to tigecycline, ceftazidime, cefepime, netilmicin, gentamicin, and meropenem. Nineteen isolates (61.3%) were tetracycline-resistant. Less than half of the isolates were resistant to colistin (9.7%), amikacin (25.8%), and trimethoprim/sulfamethoxazole (38.7%). One isolate (327) was resistant to all 21 antibiotics (Table 1).

**Table 1:** Antimicrobial susceptibility of the MDR *K. pneumoniae* isolates.

Antibiotics	MIC range (mg/L)	% R*	% I**	% S***
Cefuroxime	32–64	100.0	0.0	0.0
Cefoperazone	64	100.0	0.0	0.0
Cefazolin	16	100.0	0.0	0.0
Cefotaxime	4–8	100.0	0.0	0.0
Ciprofloxacin	8	100.0	0.0	0.0
Ampicillin	64–128	100.0	0.0	0.0
Piperacillin	128	100.0	0.0	0.0
Chloramphenicol	16–32	100.0	0.0	0.0
Ertapenem	2	100.0	0.0	0.0
Tobramycin	4–8	100.0	0.0	0.0
Aztreonam	16	100.0	0.0	0.0
Tigecycline	0.25–8	93.5	0.0	6.5
Ceftazidime	1–16	93.5	0.0	6.5
Cefepime	1–16	93.5	0.0	6.5
Netilmicin	2–16	90.3	6.5	3.2
Gentamicin	32	87.1	0.0	12.9
Meropenem	0.2516	80.6	0.0	19.4
Tetracycline	4–32	61.3	29.0	9.7
Trimethoprim/sulfamethoxazole	0.5–4	38.7	0.0	61.3
Amikacin	2–64	25.8	0.0	74.2
Colistin	0.25–16	9.7	0.0	90.3

*R, resistant; **I, intermediate; ***S, sensitive.

### 3.2 Sequence types, K- and O- loci

**Figure 1:**
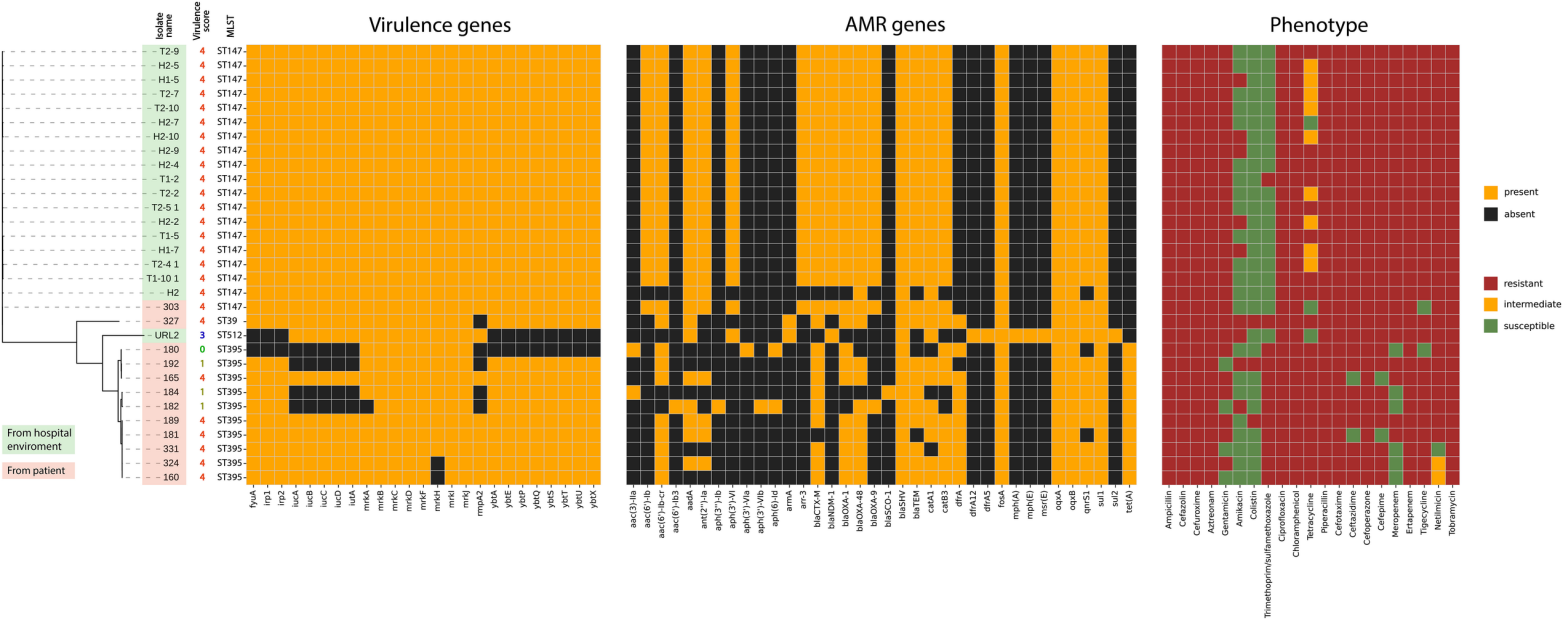
Virulence genes, virulence score, STs, antibiotic resistance profiles, and ARGs of the *K. pneumoniae* isolates.

The genome assembly quality data are shown in Supplementary Table S2. MLST analysis revealed that 10 *K. pneumoniae* isolates (31%) belonged to ST395; 19 isolates (59%) were assigned to ST147 and ST147* (a derivative of ST147 with a new allele of the *gapA* gene, 409A > G; 127Asp > Asn); and one isolate each belonged to ST39, ST443, and ST512. The main factor associated with distribution of STs was the source of the isolate, i.e., whether it was obtained from a patient or from hospital environment (Figure 1).

The isolates belonged to seven capsular types: KL64 (19; 59.4%), KL39 (5; 15.6%), KL2 (2; 6.3%), KL108 (3; 9.4%), KL23 (1; 3.1%), KL107 (1; 3.1%), and KL146 (1; 3.1%). Five ST395 isolates had a KL39 capsule locus type with an OL2α.1/O1α*β*,2α O-locus; three ST395 isolates belonged to the KL108 capsular type containing OL2α.2/O2β (*n* = 2) and OL2α.2/O1αβ,2β (*n* = 1) O-loci; and two isolates were ST395-KL2 with OL2α.1/O1αβ,2α O-loci. The 19 ST147 and ST147* isolates possessed the KL64 capsule locus and OL2α.1/O2α O-locus. The remaining three isolates were ST512-KL107 with an OL2α.2/O2β locus, ST443-KL146 with OL2α.2/O1αβ,2β, and ST39-KL23 with an OL2α.2/O1αβ,2α locus (Supplementary Table S3).

### 3.3 ARGs

Analysis of the antibiotic resistance gene (ARG) repertoire revealed that the majority (31/32) of the isolates carried between 12 and 20 ARGs, conferring resistance to β-lactams, aminoglycosides, tetracyclines, fluoroquinolones, macrolides, phenicols, sulfonamides, and trimethoprim (Figure 1; Supplementary Table S4). Resistance to ampicillin, piperacillin, cefuroxime, cefoperazone, cefazolin, cefotaxime, aztreonam, and ertapenem corresponded well with the presence of genes encoding OXA-9, OXA-1, and SHV-type beta-lactamases, as well as extended-spectrum β-lactamases (ESBLs) CTX-M and TEM, and carbapenemases (OXA-48 and NDM-1) (Hennequin and Robin, 2016).

Chloramphenicol resistance was determined by the *oqxA*, *oqxB*, *catA1*, and *catB3* genes (Shaw, 1983; Hansen et al., 2004), which were found in all MDR isolates studied. Resistance to ciprofloxacin was associated with the presence of the *aac(6′)-Ib-cr, oqxA*, *oqxB*, and *qnrS1* genes (Frasson et al., 2011). The presence of the *aac(6′)-Ib3*, *ant(2″)-Ia*, *aac(6′)-Ib-cr*, *aac(3)-IIa*, and *aac(6′)-Ib* genes corresponded well with tobramycin resistance in the MDR *K. pneumoniae* isolates (Li et al., 2023). Four *K. pneumoniae* isolates (182, 331, 160, and 192) lacked the *ant(2″)-Ia*, *aac(3)-IIa*, and *armA* genes associated with gentamicin resistance, which correlates with their susceptibility to gentamicin (Supplementary Tables S3, S4). Out of 32 isolates, 12 were resistant to trimethoprim and sulfamethoxazole and carried the *dfrA1* gene associated with trimethoprim resistance, whereas the *sul1* gene, which confers sulfamethoxazole resistance (Sköld, 2001), was found in all studied MDR *K. pneumoniae* isolates.

Three isolates (189, 192, and 327) showed resistance to colistin. Colistin resistance was shown to be determined by mutations in the *mgrB* gene or its disruption by insertion sequences (ISs) (Fordham et al., 2022; Khoshbayan et al., 2024). Among the colistin-resistant isolates, *mgrB* in isolate 327 was disrupted by an IS5-like element (L24insTer36 mutation), while isolate 189 carried the Q30Ter mutation.

Although antimicrobial resistance phenotypes and genotypes were generally correlated, some discrepancies were found. For instance, out of 32 isolates, 23 were susceptible to amikacin, of which 12 carried the *aac(6′)-Ib* gene, a determinant of amikacin resistance (Ramirez and Tolmasky, 2017; d'Udekem d'Acoz et al., 2024). Nineteen *K. pneumoniae* isolates were resistant to tetracycline, of which 10 did not carry *tet* genes, the known tetracycline resistance determinants (Grossman, 2016) (Supplementary Table S4).

### 3.4 Virulence genes

A search for virulence genes showed that all isolates carried the *mrkABCDFHIJ* genes responsible for the biosynthesis of type 3 fimbrial adhesins, except for two isolates (160 and 324) lacking the *mrkH* gene. The most isolates (29/32) contained genes controlling the biosynthesis, transport, and regulation of the yersiniabactin siderophore (*fyuA*, *irp1*, *irp2*, and *ybtAEPQSTUX*). 27 isolates carried the *iutA* and *iucABCD* genes of the *iuc1* lineage, which encode the biosynthesis of the aerobactin siderophore. In addition, 26 isolates carried the *rmpA2* gene encoding the regulator of the mucoid phenotype A, which is specific for many of hypervirulent *K. pneumoniae* isolates (Prakash et al., 2025) (Figure 1; Supplementary Table S3). According to virulence score predictions by Kleborate v3, the majority of isolates (26/32) were potentially hypervirulent (score 4).

### 3.5 Plasmids

The *K. pneumoniae* isolates harbored one to six plasmids. The most prevalent replicons were IncR (28/32), IncHI1B(pNDM-MAR) (27/32), and IncFIB(pQil) (19/32). The remaining eight replicon types ˗ Col440II, ColRNAI, ColpVC, IncFIB(K), IncFII(K), IncL, IncM1, and IncFIB(pNDM-MAR) – were detected in 2 to 5 isolates. Large hybrid plasmids with the IncHI1B(pNDM-MAR) replicon (~272–476 kb) were identified in 27 isolates. All hybrid plasmids harbored the virulence genes *iucABCD* and *iutA,* while *rmpA2* gene was detected in 26 plasmids. Twenty-five hybrid plasmids carried а *bla_OXA_* gene (Supplementary Table S3).

### 3.6 Hypermucoviscosity

According to the string test results, 28% (9/32) of *K. pneumoniae* isolates showed hypermucoviscosity phenotypes. A positive string test was observed for ST395-KL39 isolates with the OL2α.2/O2β O-locus (*n* = 4), ST395-KL2 isolates with the OL2α.1/O1αβ,2α O-locus (*n* = 2), one ST395-KL108 isolate with the OL2α.2/O2β O-locus, one ST147-KL64 isolate with the OL2α.1/O2α O-locus, and one ST512-KL107 isolate with the OL2α.2/O2β O-locus (Supplementary Table S3).

### 3.7 Mouse lethality assay

We determined the virulence level of the convergent *K. pneumoniae* isolates (*n* = 7) using a mouse lethality assay. Five isolates carried hybrid plasmids, of which three isolates (181, 189, and 331) belonged to ST395-KL39 with the OL2α.2/O2β O-locus, one isolate (H1-5) was ST147-KL64 with the OL2α.1/O2α O-locus, and one isolate (327) belonged to ST39-KL23 with the OL2α.2/O1αβ,2α O-locus. Two MDR-cKp isolates belonged to ST395-KL2 with the OL2α.1/O1αβ,2α O-locus (184), and to ST395-KL108 with OL2α.2/O2β (192) (Table 2). MDR-cKp isolate 192 and three convergent isolates with hybrid plasmids (181, 189, and 331) had LD_50_ values of 3.2 × 10^6^, 5.0 × 10^6^, 4.2 × 10^6^, and 2.72 × 10^6^ CFU/g, respectively. All these isolates except 331 showed a positive string test. The remaining three isolates, MDR-cKp 184 and convergent isolates with hybrid plasmids 327 and H1-5, had LD_50_ values of > 5.1 × 10^6^, > 4.6 × 10^6^, and > 4.0 × 10^6^ CFU/g, respectively. Among these isolates, only MDR-cKp 184 showed a positive string test.

**Table 2:** Molecular genetic characterization and LD_50_ values for *K. pneumoniae* isolates.

Isolate	ST	KL-locus	O-locus/O-type	Virulence genes in hybrid plasmids		String test	LD_50_ (CFU/g)
				*iuc1*	*rmpA2*		
181	395	KL39	OL2α.1/ O1αβ,2α	*+*	+	+	5.0 × 10^6^
184	395	KL2	OL2α.1/O1αβ,2α	*−*	−	+	> 5.1 × 10^6^
189	395	KL39	OL2α.1/ O1αβ,2α	*+*	+	+	4.2 × 10^6^
192	395	KL108	OL2α.2/ O2β	*−*	−	+	3.2 × 10^6^
331	395	KL39	OL2α.1/O1αβ,2α	*+*	+	−	2.7 × 10^6^
327	39	KL23	OL2α.2/O1αβ,2α	*+*	−	−	> 4.6 × 10^6^
H1-5	147	KL64	OL2α.1/O2α	*+*	+	−	> 4.0 × 10^6^

### 3.8 Hybrid plasmids clusters

**Figure 2:**
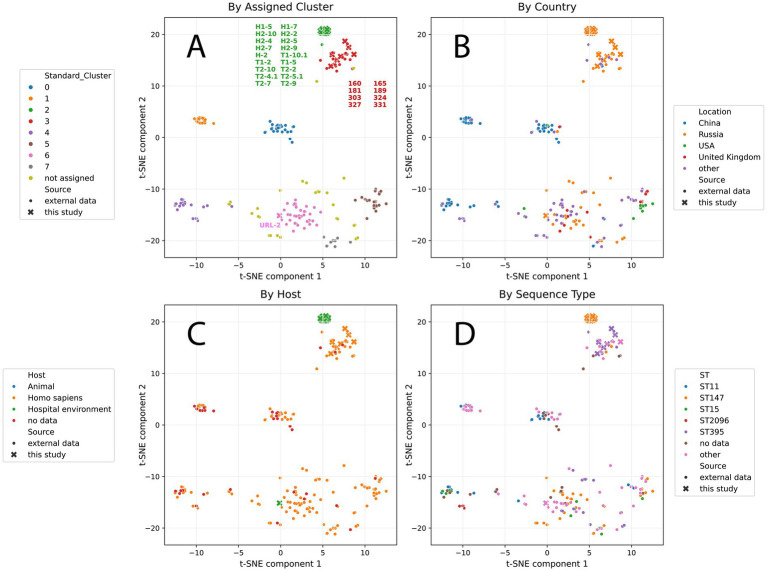
Clustering of hybrid plasmids based on their sequences and colored by clusters **(A)**, main countries of origin **(B)**, hosts **(C)**, and main sequence types (STs) **(D)**. Hybrid plasmids from *K. pneumoniae* isolates in this study are marked with cross.

Plasmids are classified as hybrids if they contain both virulence genes (such as *iuc*, *iro*, *rmpA*, and *rmpA2*) and ARGs. Hybrid plasmids (*n* = 295) were obtained from 23 countries, including Russia (37%), China (24%), the UK (7%), the USA (6%), and EU countries (9.5%). The majority of the hybrid plasmids were collected from humans (90%). The remaining hybrids plasmids were collected from hospitals environmental surfaces (8%) and animals (cow and swine; 1%) and food (1%). Cluster analysis grouped the sequences into eight clusters (Figure 2; Table 3; Supplementary Table S5).

**Table 3:** Clusters of hybrid plasmids from *K. pneumoniae.*

Cluster	*N*	Median length	Countries	Main STs	Total STs	Main replicons	Carbapenemases	Virulence genes
0	28	~293 kb	China (*n* = 22; 76%), Russia (*n* = 2), USA (*n* = 1), Taiwan (*n* = 1), Belgium (*n* = 1), United Kingdom (*n* = 1)	ST11 (*n* = 13; 56%) ST23 (*n* = 5; 17%)	7	repB (*n* = 23; 82%)	*bla_KPC-2_* (*n* = 9; 32%) *bla_NDM-1_* (*n* = 1; 3%)	*iuc1* (*n* = 25; 89.3%) *iuc1* (truncated) (*n* = 3; 10.7%) *iro1* (*n* = 10; 35.7%) *iro1* (truncated) (*n* = 4; 14.2%) *rmpA* (*n* = 21; 72%) *rmpA2* (*n* = 28; 97%)
1	21	~257 kb	China (*n* = 20; 95%), Taiwan (*n* = 1)	ST11 (*n* = 5; 28%) ST23 (*n* = 3; 18%) ST1 (*n* = 2; 11%)	12	IncFIB(K)/IncFII(K) (*n* = 18; 86%)	*bla_NDM-5_* (1; 6%) *bla_KPC-_2* (1; 6%)	*iuc1* (*n* = 1; 5%) *iuc3* (*n* = 19; 90%) *iuc3* (truncated) (*n* = 1; 5%)
2	19	~ 273 kb	Russia (*n* = 18; 96%), Ukraine (*n* = 1)	ST147 (*n* = 18; 96%)	2	IncHI1B(pNDM-MAR) (100%)	*bla_OXA-48_* (100%)	*iuc1* (*n* = 17; 89.5%) *iuc1* (truncated) (*n* = 2; 10.5%) *rmpA2* (100%)
3	42	~ 299 kb	Russia (*n* = 34; 81%), Germany (*n* = 3), Spain (*n* = 1), Switzerland (*n* = 2), China (*n* = 1), Ukraine (*n* = 1)	ST395 (*n* = 21;54%) ST39 (*n* = 7; 18%) ST147 (*n* = 4; 10%)	8	IncHI1B(pNDM-MAR) (*n* = 25; 60%) IncFIB(pNDM-Mar)/IncHI1B(pNDM-MAR) (*n* = 10; 24%)	*bla_OXA-48_* (*n* = 18; 43%)	*iuc1* (*n* = 39; 93%) *iuc1* (truncated) (*n* = 3; 7%) *rmpA2* (93%)
4	35	~ 297 kb	China (*n* = 24; 67%), India (*n* = 4), Turkey (*n* = 3), Germany *n* = (1), Taiwan (*n* = 1), Pakistan (*n* = 1)	ST11 (*n* = 13; 42%) ST2096 (*n* = 10; 32) ST15 (*n* = 7; 23%)	5	IncFIB(pNDM-Mar)/ IncHI1B(pNDM-MAR) (*n* = 19; 54%) IncFIB(K)/IncFII(K)/IncHI1B(pNDM-MAR) (*n* = 8; 23%)	*bla_KPC-2_* (*n* = 1; 3%)	*iuc1* (*n* = 34; 97.1%) *iuc 1* (truncated) (*n* = 1; 2.9%) *iro1* (*n* = 1; 2.9%) *rmpA* (*n* = 1; 4%) *rmpA2* (100%)
5	32	~ 344 kb	USA (*n* = 13; 41%), United Kingdom (*n* = 10; 31%), Egypt (*n* = 3), Australia (*n* = 1), Libya (*n* = 1), Germany (*n* = 1), Gaza Strip (*n* = 1), Lebanon (*n* = 1), Qatar (*n* = 1)	ST147 (*n* = 12; 39%) ST383 (*n* = 6; 19%) ST45 (*n* = 5; 16%)	8	IncFIB(pNDM-Mar)/IncHI1B(pNDM-MAR) (*n* = 28; 88%)	*bla_NDM-5_* (100%)	*iuc1* (100%) *rmpA* (*n* = 9; 28%) *rmpA2* (*n* = 31; 97%)
6	53	~ 348 kb	Russia (*n* = 20; 38%), United Kingdom (*n* = 8; 15%), Italy (*n* = 7; 13%), Australia (*n* = 5; 9%) Poland (*n* = 4), Ukraine (*n* = 3), Germany (*n* = 2), Croatia (*n* = 1); Czech Republic (*n* = 1), Egypt (*n* = 1)	ST147 (*n* = 21; 43%)	12	IncFIB(pNDM-Mar)/IncHI1B(pNDM-MAR) (100%)	*bla_NDM-1_* (*n* = 34, 64%)	*iuc1* (*n* = 51; 96%) *iuc1* (truncated) (*n* = 1; 2%) *iuc* unknown (*n* = 1; 2%) *rmpA* (*n* = 45; 85%) *rmpA2* (100%)
7	18	~ 309 bp	Russia (*n* = 11; 61%), China (*n* = 2), Egypt (*n* = 1), Italy (*n* = 1), Libya (*n* = 1), United Kingdom (*n* = 1), Germany (*n* = 1)	ST147 (*n* = 6; 35%) ST15 (*n* = 4; 23%) ST874 (*n* = 3; 18%)	7	IncFIB(pNDM-Mar)/IncHI1B(pNDM-MAR) (*n* = 15; 83%)	*bla_NDM-1_* (*n* = 11; 61%)	*iuc1* (*n* = 17; 94%) *iuc 1* (truncated) (*n* = 1; 6%) *rmpA* (*n* = 14; 78%) *rmpA2* (100%)
-1 (not assigned)	47	~ 357 bp	Russia (*n* = 24; 51%); Gaza Strip (*n* = 7; 15%), China (*n* = 3), USA (*n* = 3), Germany (*n* = 3), Norway (*n* = 1), Turkey (*n* = 1), Singapore (*n* = 1)	ST147 (*n* = 13; 30%) ST395 (13; 30%)	13	IncFIB(pNDM-Mar)/IncHI1B(pNDM-MAR) (25; 53%) IncFIB(pNDM-Mar)/IncHI1B(pNDM-MAR)/IncR (11; 23%)	*bla_NDM-1_* (*n* = 18; 38%) *bla_OXA-48_* (*n* = 7; 15%)	*iuc1* (*n* = 45; 96%) *iuc* unknown (*n* = 1; 2%) *iro1* (*n* = 2; 4%) *iro* unknown (*n* = 1; 2%) *rmpA* (*n* = 24; 51%) *rmpA2* (*n* = 45; 96%)

*Cluster 0* (*n* = 28) consisted mostly of the hybrid plasmids from China (*n* = 22/28). ST11 (13/29) was the dominant sequence types in this cluster. The dominant replicon was repB (*n* = 23; 82%), which co-occurred with IncHI1B(pNDM-MAR), IncFII, or IncR replicons. Nine hybrid plasmids (32%) carried the carbapenemase gene *bla_KPC-2_*. The *rmpA* and *rmpA2* genes, which regulate the mucoid phenotype, were identified in 72 and 97% of the hybrid plasmids, respectively. All hybrid plasmids carried *iuc1*. The *iro1* encoding the biosynthesis, transport, and modification of salmochelin, were found in 50% of the hybrid plasmids.

*Cluster 1* (*n* = 21) consisted of the hybrid plasmids from China (*n* = 21). The dominant sequence types were ST11 (28%), ST23 (18%), and ST1 (11%). The majority of the hybrid plasmids (82%) carried simultaneously two replicons, IncFIB(K) and IncFII(K). The carbapenemase genes *bla_KPC-2_* and *bla_NDM-5_* were identified in single isolates. The majority of the plasmids (*n* = 95) carried the *iuc3*, while the *rmpA* and *rmpA2* genes were not found.

*Cluster 2* (*n* = 19) consisted mostly of the hybrid plasmids from Russia (*n* = 18/19). The dominant sequence type was ST147. All hybrid plasmids contained one replicon, IncHI1B(pNDM-MAR), and harbored the carbapenemase gene *bla_OXA-48_*, as well as *rmpA2* gene. The *rmpA* genes were not found. All hybrid plasmids carried *iuc1*.

*Cluster 3* (*n* = 42) consisted mostly of the hybrid plasmids from Russia (*n* = 34/42). The dominant sequence types were ST395 and ST39 (29 and 18%, respectively). The majority of the hybrid plasmids carried a single replicon, IncHI1B(pNDM-MAR) (*n* = 25; 60%), and 24% of plasmids harbored two replicons, IncFIB(pNDM-Mar) and IncHI1B(pNDM-MAR). The *bla_OXA-48_* gene was found in 43% of the hybrid plasmids. The *rmpA2* gene was detected in 93% of the hybrid plasmids, whereas the *rmpA* gene were not found. All hybrid plasmids carried *iuc1* loci.

*Cluster 4* (*n* = 35) consisted mostly of the hybrid plasmids from China (*n* = 24/35). The dominant sequence types were ST11 (42%), ST2096 (32%), and ST15 (23%). The majority of the hybrid plasmids (54%) carried simultaneously two replicons - IncFIB(pNDM-Mar) and IncHI1B(pNDM-MAR). 23% of the hybrid plasmids harbored three replicons - IncFIB(K), IncFII(K), and IncHI1B(pNDM-MAR). All plasmids carried the *iuc1* and *rmpA2* gene. The carbapenemase gene *bla_KPC-2_* was detected in a single hybrid plasmid.

*Cluster 5* (*n* = 32) was geographically diverse, and consisted of the hybrid plasmids from 9 countries. The dominant sequence types were ST147 (*n* = 12; 39%) and ST383 (*n* = 6; 19%). All hybrid plasmids carried two replicons, IncFIB(pNDM-Mar) and IncHI1B(pNDM-MAR), *iuc1,* and the *bla_NDM-5_* gene. The *rmpA* and *rmpA2* genes were found in 28 and 97% of the hybrid plasmids, respectively.

Similar to *Cluster 5, Cluster 6* (*n* = 53) included hybrid plasmids collected from many countries. The majority of the hybrid plasmids were from Russia (*n* = 20; 38%). The dominant sequence type was ST147 (*n* = 21; 43%). All hybrid plasmids harbored two replicons, IncFIB(pNDM-Mar) and IncHI1B(pNDM-MAR). The *bla_NDM-1_* gene was found in 64% of plasmids. The *rmpA* and *rmpA2* genes were found in 85 and 100% of the hybrid plasmids, respectively. The *iuc1* was found in 98% of the plasmids.

*Cluster 7* (*n* = 18) included plasmids from seven countries. The dominant sequence types were ST147 (*n* = 6; 35%), ST15 (*n* = 4; 23%), and ST874 (*n* = 3; 18%). The majority of the hybrid plasmids (*n* = 15; 83%) harbored two replicons, IncFIB(pNDM-Mar) and IncHI1B(pNDM-MAR), and the *bla_NDM-1_* gene (61%). The *rmpA* and *rmpA2* genes were found in 78 and 100% of the hybrid plasmids, respectively. All hybrid plasmids carried *iuc1.*

Additionally, we checked how the identified clusters were related to metadata available for the considered strains. In general, neither host nor location demonstrated significant association with the mge-cluster results. Only hybrid plasmids from China isolates were notably prevalent in clusters 0, 1, and 5, while isolates from other countries were evenly distributed across all clusters. Unfortunately, host analysis was not informative because more than 98% of the analyzed isolates were obtained from *Homo sapiens*.

**Figure 3:**
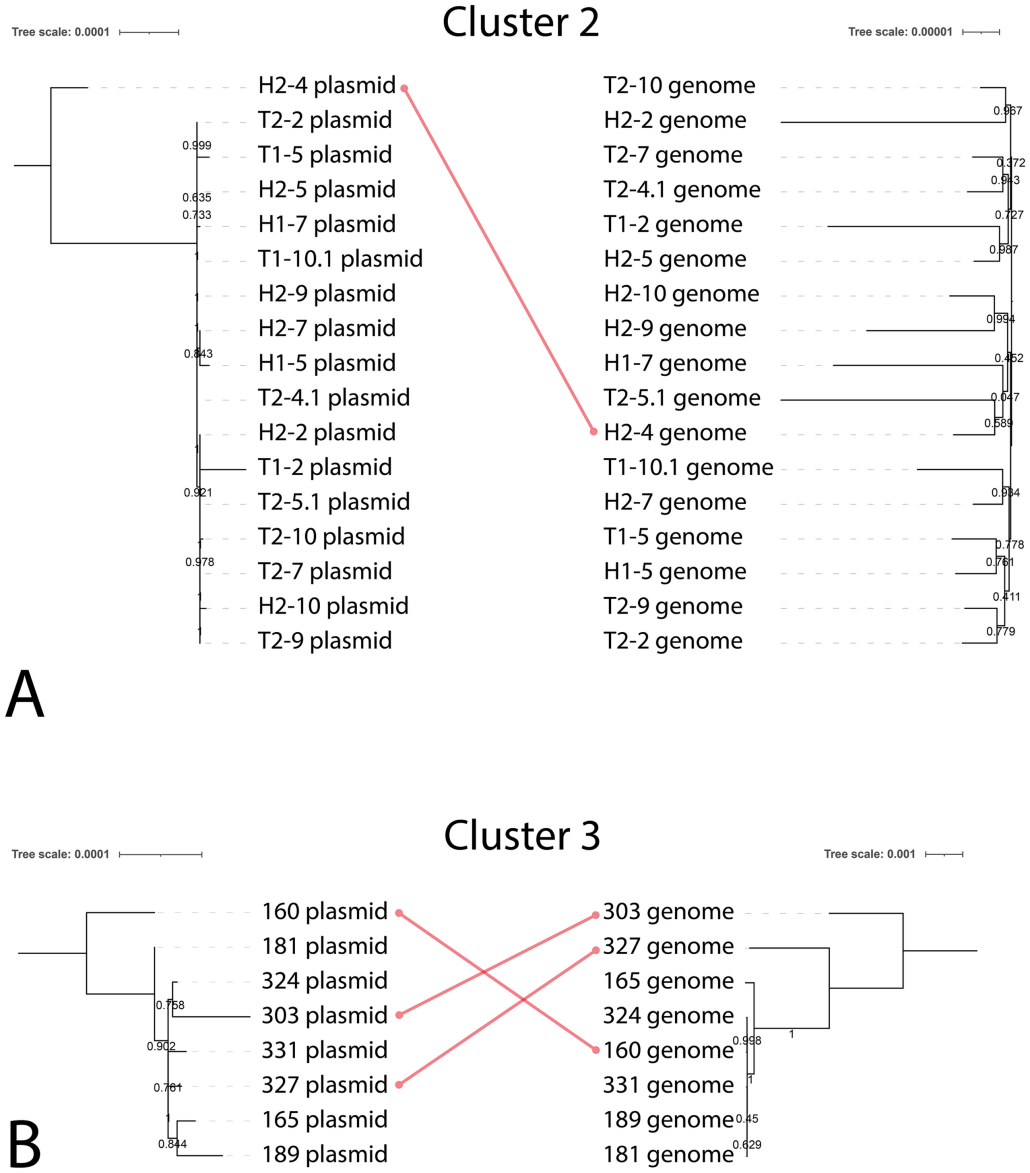
The comparison of plasmid-based and genome-based phylogenetic trees for *K. pneumoniae* strains sequenced in this study. Cluster 2 **(A)** and Cluster 3 **(B)** were chosen as comparison groups based on the mge-cluster results.

Based on the collected data, we did not identify any hybrid plasmid transmission events between hospital environment isolates and isolates from patients, as the hybrid plasmids obtained from different sources were clearly assigned to different clusters. To check if there was a plasmid transmission between hospital strains, we compared topology of phylogenetic trees constructed on whole genomes and individual hybrid plasmids from Cluster 2 (Figure 3). The resulting difference between hybrid plasmids was very consistent with the difference between whole genomes, with the exception of one plasmid from H2-4 isolate, which notably differed from other hybrid plasmids carried by the hospital environment isolates.

In the same manner, we checked spread of similar plasmids in isolates from patients (Cluster 3 on Figure 2). We identified few cases, where the leaf position in the plasmid-based tree was not consistent with that in the genome-based tree. First, the whole genome of *K. pneumoniae* strain 160 was very similar to genomes of strains 324, 331,181, and 189, but carried the plasmid from cluster 3 weakly related to others in this cluster (based on SNP number). On the other hand, *K. pneumoniae* strain 327 had sequence type different from ST395, but carried a plasmid nearly identical to that in ST395 representatives.

## 4 Discussion

*Klebsiella pneumoniae* is an opportunistic pathogen that causes a range of nosocomial infections. The emergence of convergent *K. pneumoniae* isolates, which possess features of both cKp and hvKp, is a major concern. To date, the distribution of the convergent isolates harboring hybrid plasmids has been reported in many geographical regions (Lev et al., 2018; Wyres et al., 2020; Volozhantsev et al., 2020; Lazareva et al., 2020; Banerjee et al., 2021; Starkova et al., 2021; Yong et al., 2022; Kochan et al., 2022; Arena et al., 2022; Samoilova et al., 2024; Shapovalova et al., 2024).

In this study, we report on the molecular genetic characteristics and virulence features of clinical *K. pneumoniae* isolates (*n* = 32) obtained from patients and environmental surfaces in eight hospitals across two Russian regions between 2019 and 2024. The majority of *K. pneumoniae* isolates (27/32, 84.4%) were convergent, belonging to sequence types ST147 (*n* = 19) and ST395 (*n* = 10). These linages are high-risk international clones widely spread around the world including Russia (Rodrigues et al., 2022; Arcari and Carattoli, 2023; Shaidullina et al., 2023; Edelstein et al., 2024). The high prevalence of convergent isolates among ST395 *K. pneumoniae* (85.7%) was recently revealed by an analysis of 297 genomes from 27 countries and four continents (Shaidullina et al., 2023). Similar data on the prevalence of convergent ST147 *K. pneumoniae* isolates (75.9%) were reported in the UK (Turton et al., 2024).

The most common plasmids identified in this study were IncR (28/32), IncHI1B(pNDM-MAR) (27/32), and IncFIB(pQil) (18/32). IncR plasmids are important resistance vectors that commonly carry genes conferring resistance to *β*-lactams, quinolones, aminoglycosides, sulfonamides, and tetracyclines (Zhang et al., 2025). Hybrid plasmids with IncHI1B(pNDM-MAR) replicons harbored the virulence genes *rmpA2* (*n* = 26), *iucABCD*, and *iutA* (*n* = 27). Twenty-five of these hybrid plasmids carried a *bla_OXA-48_* gene. The majority of the IncFIB(pQil) plasmids were detected in the ST147 isolates (18/19), and carried the *bla_NDM-1_* gene (17/19). Recently, ST147 *K. pneumoniae* isolates with IncFIB(pQil) plasmids with the *bla_NDM-1_* were reported in Italy (Martin et al., 2021; Di Pilato et al., 2022) and Japan (Takei et al., 2022). Furthermore, ST147 *K. pneumoniae* isolates harboring both *bla_OXA-48_* and *bla_NDM-1_* genes were identified in Germany (Sandfort et al., 2022), Italy (Nicitra et al., 2025), and in this study. The spread of convergent *K. pneumoniae* ST147 isolates co-harboring two β-lactamase genes is a concerning trend that requires monitoring to guide effective antimicrobial therapy. Phylogenetic analysis of plasmids revealed discordance between phylogenetic trees built on whole *K. pneumoniae* genomes, and trees built on individual plasmids, thus indicating potential horizontal transfer between strains isolated in one hospital. In part, we detected closely related plasmids shared by *K. pneumoniae* strains belonging to different STs. Indeed, in *K. pneumoniae*, plasmid transmission and evolution primarily occur independently of chromosome evolution (David et al., 2020).

Analysis of the *K. pneumoniae* isolates’ genomes with Kleborate v3 revealed that the majority of isolates (26/32) studied here were potentially hypervirulent (score 4) (Figure 1). All convergent *K. pneumoniae* isolates carried the aerobactin of the *iuc1* lineage and the majority (26/27) harbored the *rmpA2* gene, which has been associated with hypervirulence (Russo et al., 2015; Catalán-Nájera et al., 2017; Russo et al., 2018; Lam et al., 2018; Martin et al., 2021). While *iucABCD* loci and the *rmpA2* gene are valuable virulence indicators, the true virulence of a bacterial strain is ultimately reflected in its ability to cause disease. For this purpose, mouse models are the gold standard for identifying hvKP strains (Russo et al., 2018; Kochan et al., 2023). We determined the virulence of the convergent *K. pneumoniae* isolates (*n* = 5) in comparison to MDR-cKp isolates (*n* = 2) using a mouse lethality assay. The results of the Kleborate prediction and the mouse lethality assay were discordant. The LD_50_ for all convergent isolates carrying hybrid plasmids was >10^6^ CFU, which is typical of cKp strains and not of hvKp (Russo et al., 2024a). This low virulence may be associated with the absence of the *rmpA* genes. This is consistent with the results of a recent study which showed that deletion of the *rmpA* gene in hypervirulent *K. pneumoniae* strains of different STs increased the LD_50_ to 5.9 × 10^6^ CFU, a value comparable to that of classical *K. pneumoniae* strains (Russo et al., 2024b).

An analysis of the global population of hybrid plasmids (*n* = 295) revealed genetic diversity and mosaic structures among the hybrid plasmids in *K. pneumoniae*. Hybrid plasmids derived from *K. pneumoniae* isolates from 23 countries represented 37 different sequence types (STs). More than half of them were from isolates of ST147 (*n* = 74; 25%), ST395 (*n* = 40; 13.5%), and ST11 (*n* = 38; 13%). Overall, clustering was not linked to either ST or geographic region. For example, hybrid plasmids from ST147 *K. pneumoniae* isolates (*n* = 74) were found in six of the eight clusters and had been isolated from distinct world regions. Given that virulence and resistance determinants are typically carried by plasmid-borne mobile genetic elements, the rise of hybrid plasmids with novel mosaic architectures is unsurprising.

We could speculate that hybrid plasmids from clusters 0, 5, 6, and 7 carrying the *iuc1* locus and the *rmpA/rmpA2* genes confer a hypervirulent phenotype in *K. pneumoniae* isolates. However, several studies have demonstrated that not all isolates harboring the *rmpA* gene on hybrid plasmids exhibited a hypervirulent phenotype. For example, Martin et al. (2021) showed that convergent ST147 and ST306 *K. pneumoniae* isolates carrying IncFIB/IncHIB hybrid plasmids with the virulence genes *rmpA* and *rmpA2* were nonlethal at a challenge inoculum of 10^3^ CFU in a mouse lethality assay. Similar results were obtained by Kochan et al. (2023). Most convergent isolates had relatively low virulence levels despite containing *iuc 1, rmpA,* and *rmpA2* virulence genes. The median LD_50_ for these isolates was ~10^8^ CFU, which was comparable to MDR-cKp (Kochan et al., 2023).

In conclusion, the spread of the convergent *K. pneumoniae* isolates is increasing in many countries. It was believed that hybrid plasmids conferred a hypervirulent phenotype on *K. pneumoniae* and therefore may pose a serious public health challenge. However, as data regarding the hypervirulence of convergent isolates are contradictory, the presence of hybrid plasmids may not serve as a reliable predictor of the hypervirulent phenotype in *K. pneumoniae*. Further studies are needed to understand the genetic basis of the differences in virulence among convergent *K. pneumoniae* isolates, carrying hybrid plasmids.

## Data Availability

The datasets presented in this study can be found in online repositories. The names of the repository/repositories and accession number(s) can be found in the article/Supplementary material.

## Footnotes

1
https://www.eucast.org/

2
https://www.nih.org.pk/

4
www.bigsdb.pasteur.fr

5
https://kaptive-web.erc.monash.edu/
