# Supplementary material for: Hybrid plasmids: genetic diversity and hypervirulent phenotype of Klebsiella pneumoniae
Source: Front Microbiol. 2026 Apr 29;17:1768089. doi: 10.3389/fmicb.2026.1768089 (PMC13168089; doi:10.3389/fmicb.2026.1768089)
Supplement: Supplementary file 3 [file Table_3.DOCX]

Supplementary Table S3. Molecular genetic characterization of the *K. pneumoniae* isolates

| **Isolate** | **ST** | **KL-locus** | **O-locus/**  **O-type** | **Plasmids** | **ARGs** | | | | | **Virulence genes** | **Resistance score** | **Virulence score** | **String test (cm)** |
| --- | --- | --- | --- | --- | --- | --- | --- | --- | --- | --- | --- | --- | --- |
|  |  |  |  |  | **Carba-**  **penems** | **β-lactams** | **Aminogly-cosides** | **Fluoroqui-nolones** | **Other** |  |  |  |  |
| 160 | 395 | KL39 | OL2α.1/O1αβ,2α | Col440II  IncHI1B(pNDM-AR)/IncR | *bla_OXA-48_* | *bla_CTX-M-15_*  *bla_OXA-1_*  *bla_TEM-1B_*  *bla_SHV_* | *aac(6')-Ib-cr* | *aac(6')-Ib-cr*  *oqxA*  *oqxB*  *qnrS1* | *sul1, fosA,*  *tet(A), catA1*  *catB3, dfrA1* | *fyuA, irp1, irp2, iucABCD, iutA, rmpA2, mrkABCDFIJ,*  *ybtAEPQSTUX* | 2 | 4 | 1 |
| 165 | 395 | KL108 | OL2α.2/O2β | IncFIB(pNDM-MAR)/  IncHI1B(pNDM-MAR)  IncL  IncR | *bla_OXA-48_* | *bla_CTX-M-15_*  *bla_SHV_*  *bla_TEM-1B_*  *bla_OXA-1_* | *ant(2'')-Ia*  *aadA1*  *aac(6')-Ib-cr* | *aac(6')-Ib-cr*  *oqxA*  *oqxB*  *qnrS1* | *sul1, tet(A),*  *dfrA1, catA1*  *catB3, fosA* | *fyuA, irp1,irp2, iucABCD, iutA, rmpA2, mrkABCDFHIJ,*  *ybtAEPQSTUX* | 2 | 4 | 0 |
| 180 | 395 | KL108 | OL2α.2/O1αβ,2β | ColpVC  IncQ1  IncR | *-* | *bla_OXA-1_*  *bla_CTX-M-15_*  *bla_SHV_* | *aph(6)-Id*  *aac(6')-Ib-cr*  *aph(3')-Via*  *aac(3)-IIa* | *aac(6')-Ib-cr*  *oqxA*  *oqxB* | *sul1, fosA*  *catB3, tet(A),*  *dfrA1* | *mrkABCDFHIJ* | 1 | 0 | <0,5 |
| 181 | 395 | KL39 | OL2α.1/ O1αβ,2α | Col440II  IncHI1B(pNDM-MAR)  IncR | *bla_OXA-48_* | *bla_SHV_*  *bla_CTX-M-15_*  *bla_OXA-1_* | *aadA1*  *ant(2'')-Ia*  *aac(6')-Ib-cr* | *aac(6')-Ib-cr,*  *oqxB*  *oqxA* | *sul1, fosA, catA1*  *catB3, tet(A),*  *dfrA1* | *fyuA, irp1, irp2, iucABCD, iutA, rmpA2, mrkABCDFHIJ.*  *ybtAEPQSTUX* | 3 | 4 | 4 |
| 182 | 395 | KL2 | OL2α.1/O1αβ,2α | ColRNAI  IncFIB(pQil)/IncFII(K)  IncM1  IncR | *bla_OXA-48_* | *bla_SHV_*  *bla_TEM_*  *bla_OXA-9_*  *bla_CTX-M-14b_* | *aadA1*  *aac(6')-Ib3*  *aph(6)-Id*  *aph(3'')-Ib*  *aph(3')-VIb* | *oqxA*  *oqxB*  *qnrS1* | *fosA, catA1,*  *sul1, tet(A),*  *dfrA1* | *fyuA, irp1, irp2, mrkBCDFHIJ,*  *ybtAEPQSTUX* | 2 | 1 | 3 |
| 184 | 395 | KL2 | OL2α.1/O1αβ,2α | ColpVC  IncM1  IncR | *-* | *bla_CTX-M-3_*  *bla_SHV_*  *bla_SCO-1_*  *bla_TEM-1B_* | *aac(3)-IIa* | *oqxA*  *oqxB*  *qnrS1* | *fosA, sul1,*  *tet(A), dfrA1* | *fyuA, irp1, irp2, mrkABCDFHIJ,*  *ybtAEPQSTUX* | 1 | 1 | 4 |
| 189 | 395 | KL39 | OL2α.1/ O1αβ,2α | Col440II  IncHI1B(pNDM-MAR)/IncR | *bla_OXA-48_* | *bla_SHV_*  *bla_CTX-M-15_*  *bla_TEM-1B_*  *bla_OXA-1_* | *aadA1*  *aac(6')-Ib-cr*  *ant(2'')-Ia* | *aac(6')-Ib-cr*  *oqxA*  *oqxB*  *qnrS1* | *fosA, catA1,*  *catB3, sul1,*  *tet(A), dfrA1* | *fyuA, irp1, irp2, iucABCD, iutA, rmpA2, mrkABCDFHIJ,*  *ybtAEPQSTUX* | 3 | 4 | 4 |
| 192 | 395 | KL108 | OL2α.2/ O2β | IncL  IncR | *bla_OXA-48_* | *bla_SHV_*  *bla_CTX-M-15_*  *bla_TEM-1B_*  *bla_OXA-1_* | *aac(6')-Ib-cr* | *aac(6')-Ib-cr*  *oqxB*  *oqxA*  *qnrS1* | *fosA, catA1,*  *catB3, sul1,*  *tet(A), dfrA1* | *fyuA, irp1, irp2, mrkABCDFHIJ,*  *ybtAEPQSTUX* | 2 | 1 | 2 |
| 324 | 395 | KL39 | OL2α.1/O1αβ,2α | Col440II  ColRNAI  IncHI1B(pNDM-MAR)  IncR | *bla_OXA-48_* | *bla_SHV_*  *bla_CTX-M-15_*  *bla_TEM-1B_*  *bla_OXA-1_* | *aadA1*  *ant(2'')-Ia*  *aac(6')-Ib-cr* | *aac(6')-Ib-cr,*  *oqxB,*  *oqxA,*  *qnrS1* | *fosA, catA1,*  *catB3, sul1,*  *tet(A), dfrA1* | *fyuA, irp1, irp2, iucABCD, iutA, rmpA2, mrkABCDFIJ.*  *ybtAEPQSTUX* | 2 | 4 | 3 |
| 331 | 395 | KL39 | OL2α.1/O1αβ,2α | Col440II  IncHI1B(pNDM-MAR)  IncR | *bla_OXA-48_* | *bla_SHV_*  *bla_CTX-M-15_*  *bla_TEM-1B_*  *bla_OXA-1_* | *aac(6')-Ib-cr* | *aac(6')-Ib-cr*  *oqxB*  *oqxA*  *qnrS1* | *fosA, catB3,*  *sul1, tet(A),*  *dfrA1* | *fyuA, irp1, irp2, iucABCD, iutA, rmpA2, mrkABCDFHIJ,*  *ybtAEPQSTUX* | 2 | 4 | 0 |
| 303 | 147 | KL64 | OL2α.1/O2α | IncFIA(HI1)/IncFII(K)  IncFIB(pQil)  IncHI1B(pNDM-MAR)  IncR | *bla_OXA-48_*  *bla_NDM-1_* | *bla_SHV_*  *bla_CTX-M-15_*  *bla_OXA-1_*  *bla_TEM-1A_*  *bla_OXA-9_* | *aadA1*  *ant(2'')-Ia*  *aph(3')-VI*  *aac(6')-Ib-cr*  *aac(6')-Ib* | *aac(6')-Ib-cr*  *aac(6')-Ib*  *oqxB, oqxA*  *qnrS1* | *fosA, catA1,*  *catB3, arr-3,*  *sul1* | *fyuA, irp1, irp2, iucABCD, iutA, rmpA2, mrkABCDFHIJ.*  *ybtAEPQSTUX* | 2 | 4 | 4 |
| 327 | 39 | KL23 | OL2α.2/O1αβ,2α | ColRNAI  IncHI1B(pNDM-MAR)/IncC IncFIB(K)/IncFII(K) | *bla_OXA-48_* | *bla_SHV_*  *bla_CTX-M-55_* | *aadA1*  *armA*  *aac(6')-Ib-cr* | *aac(6')-Ib-cr,*  *oqxB*  *oqxA,*  *qnrS1* | *fosA, catA1,*  *catB3, sul1,*  *dfrA1* | *fyuA, irp1, irp2,*  *iucABCD, iutA, mrkABCDFHIJ,*  *ybtAEPQSTUX* | 3 | 4 | 0 |
| H1-5 | 147 | KL64 | OL2α.1/O2α | IncFIA(HI1)/IncFII(K)  IncFIB(pKPHS1)  IncFIB(pQil),  IncHI1B(pNDM-MAR)  IncR | *bla_OXA-48_*  *bla_NDM-1_* | *bla_SHV_*  *bla_TEM-1A_*  *bla_OXA-9_*  *bla_CTX-M-15_ bla_OXA-1_* | *aadA1 aac(6')-Ib aph(3')-VI, aac(6')-Ib-cr*  *ant(2'')-Ia* | *aac(6')-Ib-cr*  *oqxB*  *oqxA,*  *qnrS1* | *fosA, catB3,*  *catA1, arr-3,*  *sul1* | *fyuA, irp1, irp2, iucABCD, iutA, rmpA2, mrkABCDFHIJ,*  *ybtAEPQSTUX* | 2 | 4 | 0 |
| H1-7 | 147 | KL64 | OL2α.1/O2α | IncFIA(HI1)/IncFII(K)  IncFIB(pKPHS1)  IncFIB(pQil),  IncHI1B(pNDM-MAR)  IncR | *bla_OXA-48_*  *bla_NDM-1_* | *bla_SHV_*  *bla_OXA-9_*  *bla_CTX-M-15_ bla_OXA-1_*  *bla_TEM-1A_* | *aadA1*  *aac(6')-Ib*  *aph(3')-VI aac(6')-Ib-cr*  *ant(2'')-Ia* | *aac(6')-Ib-cr*  *oqxB*  *oqxA*  *qnrS1* | *fosA, catB3,*  *catA1, arr-3,*  *sul1* | *fyuA, irp1, irp2, iucABCD, iutA, rmpA2, mrkABCDFHIJ,*  *ybtAEPQSTUX* | 2 | 4 | 0 |
| H2-2 | 147* | KL64 | OL2α.1/O2α | IncFIA(HI1)/IncFII(K)  IncFIB(pKPHS1)  IncFIB(pQil)  IncHI1B(pNDM-MAR)  IncR/IncR | *bla_OXA-48_*  *bla_NDM-1_* | *bla_SHV_*  *bla_CTX-M-15_ bla_OXA-1_*  *bla_TEM-1A_*  *bla_OXA-9_* | *aadA1 aph(3')-VI aac(6')-Ib-cr aac(6')-Ib ant(2'')-Ia* | *aac(6')-Ib-cr*  *oqxB*  *oqxA*  *qnrS1* | *fosA, catB3,*  *catA1, arr-3,*  *sul1* | *fyuA, irp1, irp2, iucABCD, iutA, rmpA2, mrkABCDFHIJ,*  *ybtAEPQSTUX* | 2 | 4 | 0 |
| H2-4 | 147* | KL64 | OL2α.1/O2α | IncFIA(HI1)/IncFII(K)  IncFIB(pKPHS1)  IncFIB(pQil),  IncHI1B(pNDM-MAR)  IncR | *bla_OXA-48_*  *bla_NDM-1_* | *bla_SHV_*  *bla_CTX-M-15_ bla_OXA-9_*  *bla_OXA-1_*  *bla_TEM-1A_* | *aadA1 aac(6')-Ib ant(2'')-Ia*  *aph(3')-VI* | *oqxB*  *oqxA*  *qnrS1* | *fosA, catB3,*  *catA1, arr-3,*  *sul1 ,* | *fyuA, irp1, irp2, iucABCD, iutA, rmpA2, mrkABCDFHIJ,*  *ybtAEPQSTUX* | 2 | 4 | 0 |
| H2-5 | 147 | KL64 | OL2α.1/O2α | IncFIA(HI1)/IncFII(K)  IncFIB(pKPHS1)  IncFIB(pQil)  IncHI1B(pNDM-MAR)  IncR | *bla_OXA-48_*  *bla_NDM-1_* | *bla_SHV_*  *bla_CTX-M-15_ bla_OXA-1_*  *bla_TEM-1A_*  *bla_OXA-9_* | *aadA1 aph(3')-VI aac(6')-Ib-cr ant(2'')-Ia aac(6')-Ib* | *aac(6')-Ib-cr*  *oqxB*  *oqxA*  *qnrS1* | *fosA, catB3,*  *catA1, arr-3,*  *sul1 ,* | *fyuA, irp1, irp2, iucABCD, iutA, rmpA2, mrkABCDFHIJ,*  *ybtAEPQSTUX* | 2 | 4 | 0 |
| H2-7 | 147 | KL64 | OL2α.1/O2α | IncFIA(HI1)/IncFII(K)  IncFIB(pKPHS1)  IncFIB(pQil)  IncHI1B(pNDM-MAR)  IncR | *bla_OXA-48_*  *bla_NDM-1_* | *bla_SHV_*  *bla_CTX-M-15_*  *bla_TEM-1A_*  *bla_OXA-9_*  *bla_OXA-1_* | *aadA1 aac(6')-Ib aph(3')-VI aac(6')-Ib-cr ant(2'')-Ia* | *aac(6')-Ib-cr*  *oqxB*  *oqxA*  *qnrS1* | *fosA, catB3, catA1, arr-3, sul1* | *fyuA, irp1, irp2, iucABCD, iutA, rmpA2, mrkABCDFHIJ,*  *ybtAEPQSTUX* | 2 | 4 | 0 |
| H2-9 | 147* | KL64 | OL2α.1/O2α | IncFIA(HI1)/IncFII(K)  IncFIB(pKPHS1)  IncFIB(pQil)  IncHI1B(pNDM-MAR)  IncR | *bla_OXA-48_*  *bla_NDM-1_* | *bla_SHV_*  *bla_CTX-M-15_*  *bla_TEM-1A_*  *bla_OXA-9_*  *bla_OXA-1_* | *aadA1,*  *aac(6')-Ib aph(3')-VI aac(6')-Ib-cr*  *ant(2'')-Ia* | *aac(6')-Ib-cr*  *oqxB*  *oqxA*  *qnrS1 (2)* | *fosA, catB3, catA1, arr-3,*  *sul1* | *fyuA, irp1, irp2, iucABCD, iutA, rmpA2, mrkABCDFHIJ,*  *ybtAEPQSTUX* | 2 | 4 | 0 |
| H2-10 | 147 | KL64 | OL2α.1/O2α | IncFIA(HI1)/IncFII(K)  IncFIB(pQil)  IncHI1B(pNDM-MAR)  IncR | *bla_OXA-48_*  *bla_NDM-1_* | *bla_SHV_*  *bla_CTX-M-15_ bla_TEM-1A_*  *bla_OXA-9_*  *bla_OXA-1_* | *aadA1 (2)*  *aac(6')-Ib aph(3')-VI aac(6')-Ib-cr*  *ant(2'')-Ia* | *aac(6')-Ib-)*  *oqxB*  *oqxA*  *qnrS1* | *fosA, catB3,*  *catA1, arr-3,*  *sul1* | *fyuA, irp1, irp2, iucABCD, iutA, rmpA2, mrkABCDFHIJ,*  *ybtAEPQSTUX* | 2 | 4 | 0 |
| T1-5 | 147* | KL64 | OL2α.1/O2α | IncFIA(HI1)/IncFII(K)  IncFIB(pKPHS1)/  IncFIB(pKPHS1)  IncFIB(pQil)  IncHI1B(pNDM-MAR)  IncR | *bla_OXA-48_*  *bla_NDM-1_* | *bla_SHV_*  *bla_CTX-M-15_ bla_TEM-1A_*  *bla_OXA-1_*  *bla_OXA-9_* | *aadA1*  *aph(3')-VI aac(6')-Ib-cr*  *aac(6')-Ib ant(2'')-Ia* | *aac(6')-Ib-cr*  *oqxB*  *oqxA*  *qnrS1* | *fosA, catB3,*  *catA1, arr-3,*  *sul1* | *fyuA, irp1, irp2, iucABCD, iutA, rmpA2, mrkABCDFHIJ,*  *ybtAEPQSTUX* | 2 | 4 | 0 |
| T1-10.1 | 147* | KL64 | OL2α.1/O2α | IncFIA(HI1)/IncFII(K)  IncFIB(pKPHS1)  IncFIB(pQil)  IncHI1B(pNDM-MAR)  IncR | *bla_OXA-48_*  *bla_NDM-1_* | *bla_SHV_*  *bla_CTX-M-15_ bla_TEM-1A_*  *bla_OXA-1_*  *bla_OXA-9_* | *aadA1*  *aph(3')-VI aac(6')-Ib-cr aac(6')-Ib ant(2'')-Ia* | *aac(6')-Ib-cr*  *oqxB*  *oqxA*  *qnrS1* | *fosA, catB3, catA1, arr-3,*  *sul1* | *fyuA, irp1, irp2, iucABCD, iutA, rmpA2, mrkABCDFHIJ,*  *ybtAEPQSTUX* | 2 | 4 | 0 |
| T2-2 | 147* | KL64 | OL2α.1/O2α | IncFIA(HI1)/IncFII(K)  IncFIB(pKPHS1)  IncFIB(pQil)  IncHI1B(pNDM-MAR)  IncR | *bla_OXA-48_*  *bla_NDM-1_* | *bla_SHV_*  *bla_CTX-M-15_ bla_TEM-1A_*  *bla_OXA-1_*  *bla_OXA-9_* | *aadA1*  *aph(3')-VI aac(6')-Ib-cr ant(2'')-Ia aac(6')-Ib* | *aac(6')-Ib-cr*  *oqxB*  *oqxA*  *qnrS1* | *fosA, catB3, catA1, arr-3,*  *sul1* | *fyuA, irp1, irp2, iucABCD, iutA, rmpA2, mrkABCDFHIJ,*  *ybtAEPQSTUX* | 2 | 4 | 0 |
| T2-4.1 | 147 | KL64 | OL2α.1/O2α | IncFIA(HI1)/IncFII(K)  IncFIB(pKPHS1)  IncFIB(pQil)  IncFII(K)  IncHI1B(pNDM-MAR)  IncR | *bla_OXA-48_*  *bla_NDM-1_* | *bla_SHV_*  *bla_CTX-M-15_ bla_TEM-1A_*  *bla_OXA-1_*  *bla_OXA-9_* | *aadA1 (3)*  *aph(3')-VI aac(6')-Ib-cr*  *aac(6')-Ib ant(2'')-Ia* | *aac(6')-Ib-cr*  *oqxB*  *oqxA*  *qnrS1* | *fosA, catB3,*  *catA1, arr-3,*  *sul1* | *fyuA, irp1, irp2, iucABCD, iutA, rmpA2, mrkABCDFHIJ,*  *ybtAEPQSTUX* | 2 | 4 | 0 |
| T2-5.1 | 147* | KL64 | OL2α.1/O2α | IncFIA(HI1)/IncFII(K)  IncFIB(pKPHS1)  IncFIB(pQil)  IncHI1B(pNDM-MAR)  IncR/IncR | *bla_OXA-48_*  *bla_NDM-1_* | *bla_SHV_*  *bla_TEM-1A_*  *bla_CTX-M-15_ bla_OXA-1_*  *bla_OXA-9_* | *aadA aph(3')-VI aac(6')-Ib-cr ant(2'')-Ia aac(6')-Ib* | *aac(6')-Ib-cr*  *oqxB*  *oqxA*  *qnrS1* | *fosA, catB3,*  *catA1, arr-3,*  *sul1* | *fyuA, irp1, irp2, iucABCD, iutA, rmpA2, mrkABCDFHIJ,*  *ybtAEPQSTUX* | 2 | 4 | 0 |
| T2-7 | 147 | KL64 | OL2α.1/O2α | IncFIA(HI1)/IncFII(K)  IncFIB(pKPHS1)  IncFIB(pQil)  IncHI1B(pNDM-MAR)  IncR | *bla_OXA-48_*  *bla_NDM-1_* | *bla_SHV_*  *bla_TEM-1A_*  *bla_OXA-9_*  *bla_CTX-M-15_ bla_OXA-1_* | *aadA1 ant(2'')-Ia aph(3')-VI aac(6')-Ib-cr aac(6')-Ib* | *aac(6')-Ib-cr*  *oqxB*  *oqxA*  *qnrS1* | *fosA, catB3,*  *catA1, arr-3,*  *sul1* | *fyuA, irp1, irp2, iucABCD, iutA, rmpA2, mrkABCDFHIJ,*  *ybtAEPQSTUX* | 2 | 4 | 0 |
| T2-9 | 147* | KL64 | OL2α.1/O2α | IncFIA(HI1)/IncFII(K)  IncFIB(pKPHS1)  IncFIB(pQil)  IncHI1B(pNDM-MAR)  IncR | *bla_OXA-48_*  *bla_NDM-1_* | *bla_SHV_*  *bla_TEM-1A_*  *bla_OXA-9_*  *bla_CTX-M-15_ bla_OXA-1_* | *aadA1*  *ant(2'')-Ia*  *aac(6')-Ib*  *aph(3')-VI aac(6')-Ib-cr* | *aac(6')-Ib-cr*  *oqxB*  *oqxA*  *qnrS1* | *fosA, catB3,*  *catA1, arr-3,*  *sul1* | *fyuA, irp1, irp2, iucABCD, iutA, rmpA2, mrkABCDFHIJ,*  *ybtAEPQSTUX* | 2 | 4 | 0 |
| T2-10 | 147 | KL64 | OL2α.1/O2α | IncFIA(HI1)/IncFII(K)  IncFIB(pKPHS1)  IncFIB(pQil)  IncHI1B(pNDM-MAR)  IncR | *bla_OXA-48_*  *bla_NDM-1_* | *bla_SHV_*  *bla_TEM-1A_*  *bla_OXA-9_*  *bla_CTX-M-15_ bla_OXA-1_* | *aadA1*  *aph(3')-VI aac(6')-Ib-cr*  *ant(2'')-Ia aac(6')-Ib* | *aac(6')-Ib-cr*  *oqxB*  *oqxA*  *qnrS1* | *fosA, catB3,*  *catA1, arr-3,*  *sul1* | *fyuA, irp1, irp2, iucABCD, iutA, rmpA2, mrkABCDFHIJ,*  *ybtAEPQSTUX* | 2 | 4 | 0 |
| T1-2 | 147* | KL64 | OL2α.1/O2α | IncFIA(HI1)  IncFIB(pKPHS1)  IncFIB(pQil)  IncFII(K)  IncHI1B(pNDM-MAR)  IncR | *bla_OXA-48_*  *bla_NDM-1_* | *bla_SHV_*  *bla_TEM-1A_*  *bla_OXA-9_*  *bla_CTX-M-15_ bla_OXA-1_* | *aadA1*  *aph(3')-VI aac(6')-Ib-cr*  *ant(2'')-Ia*  *aac(6')-Ib* | *aac(6')-Ib-cr*  *oqxB*  *oqxA*  *qnrS1* | *fosA, catB3,*  *catA1, arr-3,*  *sul1* | *fyuA, irp1, irp2, iucABCD, iutA, rmpA2, mrkABCDFHIJ,*  *ybtAEPQSTUX* | 2 | 4 | 0 |
| H-2 | 147 | KL64 | OL2α.1/O2α | IncFIA(HI1)/IncFII(K)  IncFIB(pKPHS1)  IncHI1B(pNDM-MAR) | *bla_OXA-48_* | *bla_SHV_* | *aadA1*  *ant(2'')-Ia* | *oqxB*  *oqxA* | *fosA,catA1,*  *sul1* | *fyuA, irp1, irp2, iucABCD, iutA, rmpA2, mrkABCDFHIJ,*  *ybtAEPQSTUX* | 2 | 4 | 0 |
| URL-2 | 512 | KL107 | OL2α.2/O2β | IncFIB(K)/IncFII(K)  IncFIB(pKPHS1)  IncFIB(pNDM-Mar)/ IncHI1B(pNDM-MAR) | *bla_NDM-1_* | *bla_SHV_* | *aph(3')-VI*  *armA*  *aadA2* | *oqxB*  *oqxA*  *qnrS1* | *fosA6, msr(E), mph(A), mph(E),*  *sul1, sul2, dfrA5, dfrA12, catA1* | *iucABCD,*  *iutA,*  *mrkABCDFHIJ,*  *rmpA2* | 2 | 3 | 1 |
| CIB-6 | 443 | KL146 | OL2α.2/O1αβ,2β | IncFIB(K) | *-* | *bla_SHV_* | *-* | *oqxB*  *oqxA* | *fosA6* | *mrkABCDFHIJ* | 0 | 0 | 0 |
